# B cells: roles in physiology and pathology of pregnancy

**DOI:** 10.3389/fimmu.2024.1456171

**Published:** 2024-10-07

**Authors:** Jin-Chuan Liu, Qunxiong Zeng, Yong-Gang Duan, William S. B. Yeung, Raymond H. W. Li, Ernest H. Y. Ng, Ka-Wang Cheung, Qingqing Zhang, Philip C. N. Chiu

**Affiliations:** ^1^ Department of Obstetrics and Gynaecology, School of Clinical Medicine, Li Ka Shing (LKS) Faculty of Medicine, The University of Hong Kong, Hong Kong, Hong Kong SAR, China; ^2^ Shenzhen Key Laboratory of Fertility Regulation, The University of Hong Kong-Shenzhen Hospital, Shenzhen, China

**Keywords:** B cells, pregnancy, maternal-fetal interface, immune tolerance, pregnancy complications

## Abstract

B cells constitute a diverse and adaptable immune cell population with functions that can vary according to the environment and circumstances. The involvement of B cells in pregnancy, as well as the associated molecular pathways, has yet to be investigated. This review consolidates current knowledge on B cell activities and regulation during pregnancy, with a particular focus on the roles of various B cell subsets and the effects of B cell-derived factors on pregnancy outcomes. Moreover, the review examines the significance of B cell-associated autoantibodies, cytokines, and signaling pathways in relation to pregnancy complications such as pregnancy loss, preeclampsia, and preterm birth.

## 1 Introduction

Pregnancy poses a considerable immunological challenge as a mother must support her baby with half of genetic makeup different from hers. A successful pregnancy depends on the intricate interaction between the fetal placenta, and the maternal decidua at the maternal-fetal interface. Throughout pregnancy, the immune cells at the maternal-fetal interface create an environment of immune tolerance. This not only protects the fetus from the maternal immune system but also offers defense for the mother against pathogens. The decidua contains various immune cell populations. Among the immune cells, natural killer (NK) cells, macrophages, T cells and dendritic cells are the prevalent leukocytes (1, 2). They not only prevent immune rejection of the fetus but also support trophoblast invasion, tissue remodeling, and angiogenesis to establish a healthy placenta (2–4). Disruption of the immune microenvironment at the maternal-fetal interface as often caused by inflammation can result in adverse pregnancy outcomes such as preterm labor, preeclampsia (PE), fetal growth restriction, or pregnancy loss. Inflammatory responses can be triggered by infections, autoimmune diseases, stress, or other unknown factors.

B cells in humans and mice originate from hematopoietic stem cells (HSCs) and mature in the fetal liver and bone marrow (5, 6). They play a significant role in both humoral and cellular immunity. In humoral immunity, they produce specific antibodies in response to infections or vaccinations. In cellular immunity, they activate T cells by presenting antigens, offering co-stimulation signals, and generating cytokines (7, 8). Since their discovery over a century ago, scientists have identified several subsets of B cells, including B1 and B2 lymphocytes. The latter are further divided into marginal zone (MZ) and follicular (FO) B cells (8).

Although B cells account for only 1–2% of the decidual lymphocytes, their importance in promoting a healthy pregnancy is gaining attention (9) due to their ability to produce antibodies as well as other cell regulatory factors. The proportion of decidual B cells modestly increased between 27 and 33 weeks of gestation followed by a slight decline at term (10). In mice, a lack of mature B cells correlates with reduced litter size, smaller embryos, and heightened susceptibility to prenatal infections (11). In women with common variable immunodeficiency, a condition characterized by reduced levels of switched memory B cells (12), there is an increased risk for preterm birth (PTB) and other pregnancy complications, including PE, stillbirth, and vaginal bleeding (13). Both recurrent pregnancy loss (RPL) and PE have been linked to alterations in the development of B cell memory (14–16).

Current research suggests that during pregnancy, B cells create a tolerant environment by generating protective antibodies against foreign paternal antigens. In addition, a subset of interleukin 10 (IL-10) secreting regulatory B cells (Bregs) is important for establishing an anti-inflammatory microenvironment to sustain pregnancy (17). However, B cells can also negatively affect pregnancy by producing autoantibodies that target the mother’s tissues and complicate pregnancy. For example, these autoantibodies may attack the placenta, hindering its functionality and causing PE, RPL, or intrauterine fetal death. Consistently, certain autoimmune diseases exacerbated by these autoantibodies, such as systemic lupus erythematosus (SLE), can increase the risk of RPL, PTB, and other adverse pregnancy outcomes (18–23). Despite their significance, research on B cells in normal and complicated pregnancies has been largely neglected, making this area underexplored in the field.

## 2 B cell development and differentiation

### 2.1 B cell development and function

**Figure 1:**
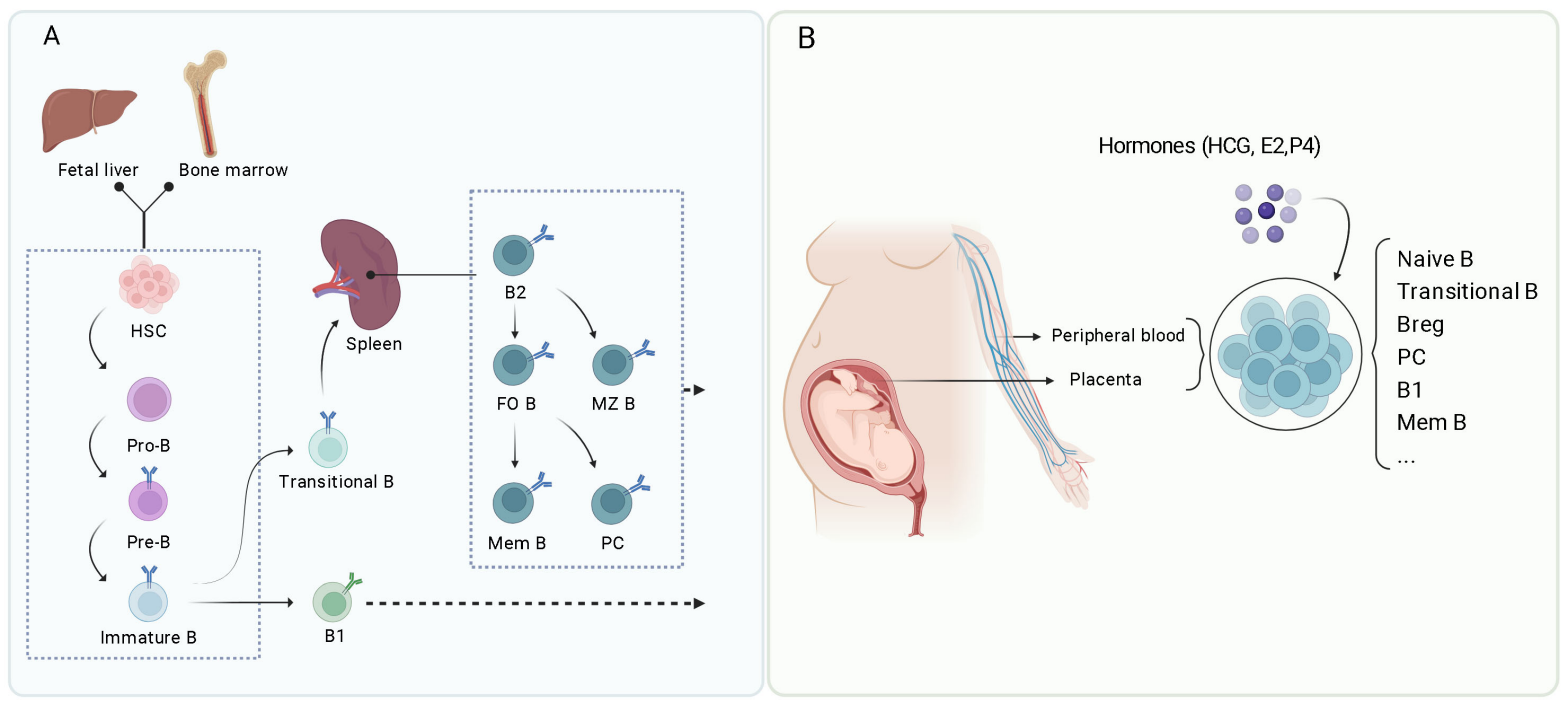
(Created with BioRender.com): Overview of B cell development and differentiation. **(A)** B cells develop from hematopoietic stem cells (HSC) in the fetal liver and bone marrow, leading to the formation of various B cell subsets including B1 cells, B2 cells (including follicular B cells or FO B, marginal zone B cells or MZ B), memory B cells (Mem B), and plasma cells (PC). **(B)** During pregnancy, various B cell subsets play important roles in maintaining a health environment, including naïve B cells, transitional B cells, Bregs, plasma cells, B1 B cells, and memory B cells. These subsets are regulated by hormones such as human chorionic gonadotropin (hCG), estradiol (E2), and progesterone (P4).

B cells originate from HSCs. They undergo a tightly regulated developmental process to ensure their functionality and avoid self-reactivity. The B1 cells primarily develop in the fetal liver, while the B2 cells emerge from the bone marrow (**Figure 1A**) (8, 24). B cell differentiation in the bone marrow consists of several key stages: early stage, pro-B cells, pre-B cells, and immature B cells (**Figure 1A**). The process begins with HSCs differentiation into common lymphoid progenitors. Those committed to the B cell lineage become pro-B cells characterized by expression of specific surface markers and immunoglobulin (Ig) gene rearrangement (8). In humans, pro-B cells express CD19, a crucial B cell marker, along with other markers such as CD34 and CD45R (B220). Successful IgH gene rearrangement enables the pro-B cells to advance to the pre-B cell stage, during which light chain gene rearrangement occurs.

The pre-B cells express the pre-B cell receptor (pre-BCR), which consists of the antibody’s heavy chain (μH), surrogate light chains, and signaling molecules Igα and Igβ (25). Upon successful light chain rearrangement, the cells display the B cell receptor (BCR) on their surface and become immature B cells. These cells have a strong affinity for self-antigens and undergo negative selection, eliminating self-reactive B cells through central tolerance.

The immature B cells migrate to peripheral lymphoid organs, such as the spleen, where they develop into transitional B cells before reaching full maturity. In the spleen, the transitional B cells receive signals from their BCR and other surface molecules within the surrounding microenvironment, including interactions with follicular dendritic cells and T cells. The combination of these interactions and survival signals, such as B-cell activating factor (BAFF), facilitate the development of transitional B cells into two distinct types: FO B cells and MZ B cells. FO B cells are predominantly located in the follicles of secondary lymphoid organs and play a crucial role in producing high-affinity antibody responses. MZ B cells are situated in the spleen’s marginal zone, specializing in rapid reactions to pathogens in the bloodstream. Both FO and MZ B cells are fully mature B cells that can respond to antigens effectively (**Figure 1A**).

Upon encountering antigens, mature FO B cells further differentiate into memory B cells or antibody-secreting plasma cells. This differentiation involves germinal center (GC) reactions and is influenced by T follicular helper cells (Tfh) (8). Tfh cells are a subset of T cells that play a significant role in the formation of GCs within lymphoid tissues (26). These GCs facilitate B cell proliferation, differentiation, and somatic hypermutation (SHM). Following antigen detection, FO B cells increase the expression of chemokine receptors, such as C-C chemokine receptor 7 (CCR7) and Epstein-Barr virus-induced receptor 2. This upregulation enables FO B cells to move to the T-B border, a region adjacent to the T-cell zone within the follicle. At the T-B border, FO B cells interact with antigen-specific Tfh cells, which provide crucial costimulatory signals, such as CD40L, and cytokines like interleukin 4 (IL-4), interleukin 21 (IL-21), and interferon gamma (IFN-γ). These factors contribute to B cell proliferation and differentiation. A portion of activated FO B cells increases B-cell lymphoma 6 (Bcl6) expression and returns to the follicles to establish GCs alongside Tfh cells. Within GCs, B cells experience extensive proliferation, SHM, and class switch recombination (CSR). The SHM process introduces mutations into immunoglobulin genes, thereby improving the affinity of the resulting antibodies. Meanwhile, CSR alters the antibody isotype, enabling it to perform various effector functions. High-affinity B cells that successfully compete for antigens in the GC and receive sufficient Tfh signals differentiate into memory B cells, which can endure long durations and quickly react to future exposures of the same antigen. Additionally, some B cells differentiate into long-lived plasma cells that migrate to the bone marrow and secrete substantial amounts of high-affinity antibodies (8, 26).

### 2.2 B cell receptor signaling and activation

BCR signaling is essential for B cell activation, differentiation, and immune response. It begins when the BCR on the surface of B cells binds to a specific antigen (27). The BCR complex consists of a membrane-bound immunoglobulin molecule (mIg) that recognizes antigens and a signaling module composed of Igα and Igβ chains (28, 29). These components collaborate to transmit signals into the cell, leading to B cell activation, differentiation, and antibody production. Upon binding with the antigens, the BCRs cluster together and initiate a series of intracellular signaling events, involving the recruitment and activation of various signaling molecules, such as kinases like Lyn and Syk, adaptors such as B cell linker protein (BLNK), and enzymes like phosphoinositide 3-kinase (PI3K) (29). This results in the activation of downstream signaling molecules and transcription factors. The signaling cascade increases intracellular calcium levels and activates nuclear factors such as nuclear factor kappa B (NFκB) and nuclear factor of activated T cells (NFAT) (29). These factors move to the nucleus and drive the transcription of genes vital for B cell activation and function. To achieve full activation and differentiation into antibody-producing plasma cells or memory B cells, B cells require a second signal, provided by helper T cells through CD40L-CD40 interactions or by pattern-recognition receptors recognizing pathogen-associated molecular patterns (PAMPs) (29).

Proper BCR signaling is crucial for protective immunity but can contribute to pathological conditions if dysregulated. For instance, BCR signaling dysregulation can cause autoimmune diseases or B cell malignancies (30, 31), while defective BCR signaling can lead to immunodeficiencies (32, 33). During pregnancy, BCR develops a diverse repertoire through processes such as somatic recombination, class switch recombination, and somatic hypermutation. This diversity enables the fetus to establish a stable immune response (34, 35). The repertoire is achieved by selecting and rearranging variable (V), diversity (D), and joining (J) gene segments, as well as random nucleotide insertions and deletions facilitated by terminal deoxynucleotidyl transferase (TdT) (35). BCR activity is also modulated to enhance the functions of Bregs, which help to prevent maternal immune responses against the fetus by producing anti-inflammatory cytokines (36). Moreover, maternal B lymphocytes with BCRs specific for paternal antigens or trophoblast giant cells trigger the deletion of these B cells in the spleen and bone marrow, beginning in mid-pregnancy and reversing after childbirth. This mechanism aids in preventing maternal immune rejection of the semi-allogeneic fetus by reducing the population of B cells that could recognize and attack fetal antigens (37, 38).

## 3 Localization of B cells in the maternal-fetal interface

B cells are found in the decidua basalis, decidua parietalis, and placental tissue within the interface. They are present in the human term decidua (39, 40); the number of B cells is higher in the term decidua basalis than in the decidua parietalis tissues, and the decidua parietalis has a greater proportion of mature/naive B cells while the decidua basalis has more transitional B cells (40). During preterm labor and placental inflammation, the total B cell count increases and the proportion of various B cell subsets changes in human choriodecidua (39). The functional role of B cells in the decidua parietalis of pregnant women remains to be investigated.

Decidual B cells express high levels of interleukin 12 (IL-12), interleukin 6 (IL-6), and interleukin 35 (IL-35). The number of B1 cells and plasmablasts (a short-lived, antibody-secreting cells that are an intermediate stage between activated B cells and fully differentiated plasma cells) also increases in women experiencing labor and chronic chorioamnionitis. The observed increase in immune memory and the consistent presence of B cells at the fetal-maternal interface suggest that B cells may be a normal decidual component of a healthy pregnancy rather than indicators of uterine abnormalities. In this context, the decidual B cells produce IL-10 and are found in clusters alongside the Foxp3^+^ T cells (41). Further research is necessary to clarify the role of B cells in pregnancy.

B cells have been found in the chorionic villi and the decidua (42–44). The immune landscape in the villous placenta is distinct from the choriodecidua, characterized by a significant population of monocytes, including fetal-origin Hofbauer cells, and a diverse immune cell population such as T cells, monocytes, dendritic cells, NK cells, neutrophils, and B cells (44). The B cells are also found within the trophoblast boundary and beyond fetal blood vessels in the second-trimester placenta (43). Advanced single-cell techniques have identified B cells in healthy placentas from women who delivered at term, with or without spontaneous labor (42, 45). In full-term healthy pregnancies, the mature/naïve B cells are retained in placental intervillous blood due to specific placenta-produced chemokines (46), indicating the potential role of these cells in fetal immune responses. The presence of B cells in both maternal and fetal compartments is consistent with their involvement in immune interactions at the maternal-fetal interface.

There is currently no evidence of the presence of B cells in the fetal membranes. However, extracts from fetal membranes during labor induce greater leukocyte chemotaxis than those from non-labor membranes, implying that B cells and other leukocytes may be attracted to the fetal membranes during labor (47). Furthermore, the pre-B-cell colony-enhancing factor (PBEF), known to play a role in the maturation of B cell precursors, is consistently expressed in the fetal membranes and placenta, with increased expression during labor (48, 49). These findings suggest that the role of B cells in the immune environment of fetal membranes and their impact on pregnancy outcomes is a potential research area.

## 4 Role of B cells at the maternal-fetal interface

### 4.1 B cells in antigen presentation

**Figure 2:**
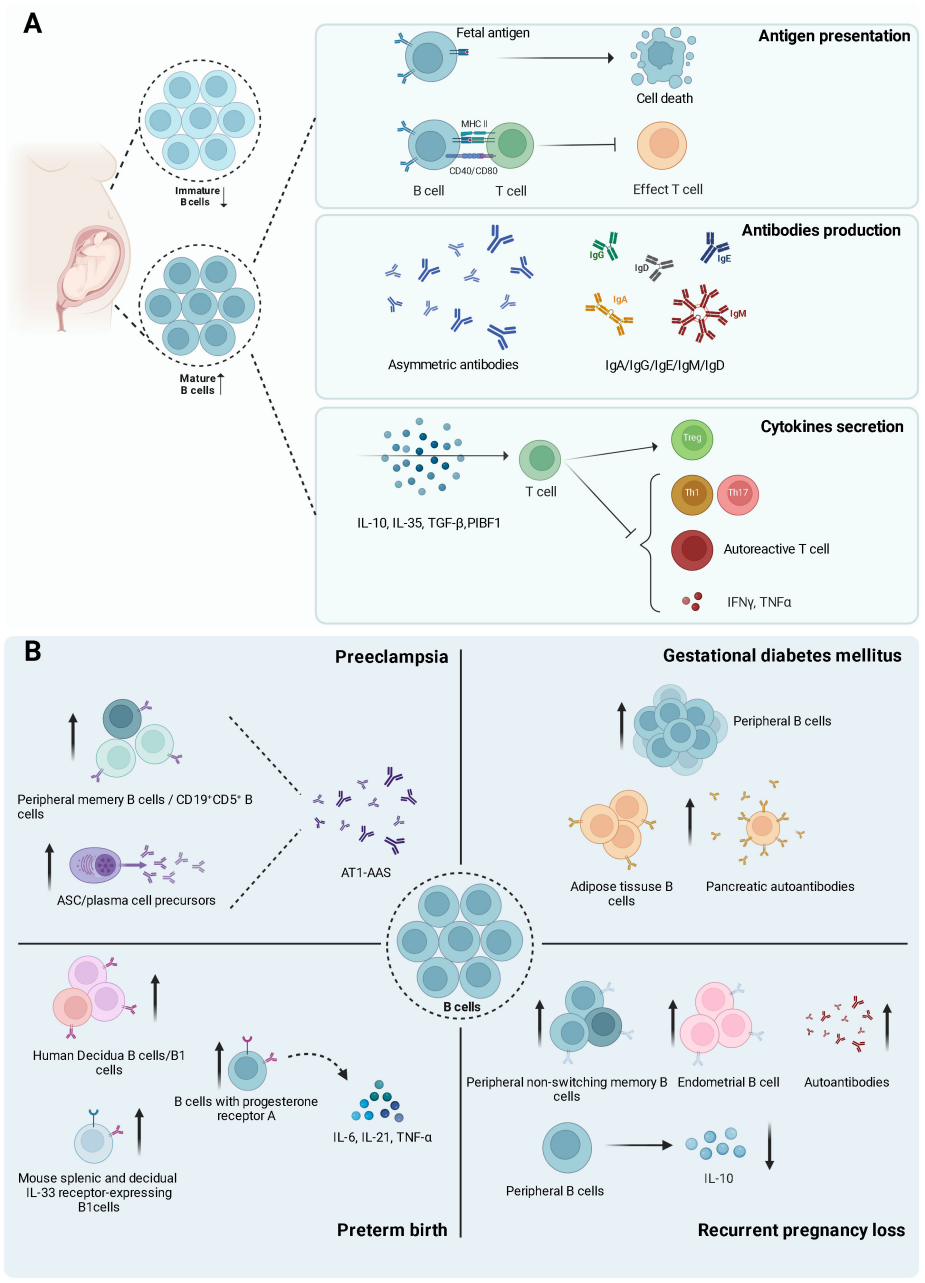
(Created with BioRender.com): Diverse functions of B cells during normal pregnancy and pregnancy-related disorders. **(A)** The Multifaceted Roles of B Cells During Pregnancy. The left panel illustrates the altered B cell populations during pregnancy, characterized by decreased immature B cells and increased mature B cells. The right panel delineates three primary functions of B cells: 1) Antigen Presentation: B cells encountering fetal antigens may undergo deletion, potentially eliminating autoreactive B cells. Additionally, B cells present antigens to T cells via MHC II, leading to the inhibition of effector T cell differentiation. 2). Antibody Production: B cells generate a diverse repertoire of antibodies during pregnancy, including asymmetric antibodies and various immunoglobulin isotypes (IgA, IgG, IgE, IgM, and IgD). 3) Cytokine Secretion: B cell-derived cytokines (IL-10, IL-35, TGF-β, and PIBF1) exert immunomodulatory effects on T cell subsets. These cytokines promote the differentiation of regulatory T cells (Treg) while suppressing Th1, Th17, and autoreactive T cells that produce pro-inflammatory cytokines such as IFN-γ and TNF-α. **(B)** The diverse roles and alterations of B cells in four pregnancy-related complications are as follows: 1) Preeclampsia: Increased peripheral memory B cells, CD19+CD5+ B cells, and antibody-secreting cell (ASC)/plasma cell precursors. These cells contribute to the production of AT1-AAS (Angiotensin II Type 1 Receptor Agonistic Autoantibodies), implicated in preeclampsia pathogenesis. 2) Gestational Diabetes Mellitus: Elevated peripheral and adipose tissue B cells, accompanied by increased pancreatic autoantibodies, suggesting a potential autoimmune component in its development. 3) Preterm Birth: Increased human decidual B cells/B1 cells, B cells expressing progesterone receptor A, and murine splenic and decidual IL-33 receptor-expressing B1 cells. B cells expressing progesterone receptor A are associated with enhanced production of pro-inflammatory cytokines (IL-6, IL-21, TNF-α), potentially contributing to preterm birth. 4) Recurrent Pregnancy Loss: Increased peripheral non-switching memory B cells, endometrial B cells, and autoantibodies. Conversely, there is a decrease in peripheral B cells producing the anti-inflammatory cytokine IL-10.

B cells process and present antigens in association with either MHC class I or MHC class II molecules to CD8 and CD4 cells, respectively, ultimately leading to the activation of the adaptive immune response (50). Antigen presentation is crucial for enabling a targeted immunogenic response from the immune system. The fetal-placental antigens that interact with the maternal immune system include blood group antigens, MHC antigens, and minor histocompatibility antigens. Despite being significantly different from the mother, these antigens do not elicit the robust cellular or humoral immune reactions typically seen following organ transplantation (51). Previous research suggests that B cells specific to the fetal antigens are eliminated during pregnancy to maintain immune tolerance at the maternal-fetal interface. When the MHC class I molecule, specifically H-2Kb, is expressed in mouse trophoblast giant cells, it is recognized by the maternal immune system and triggers a response in the maternal bone marrow, leading to the deletion of a B cell subpopulation, including immature and transitional B cells (**Figure 2A**). This deletion is essential for developing maternal tolerance towards the fetus (38). Recent research has also shown that B cells play a vital role in mediating the MHC-class-II-restricted presentation of antigens to CD4^+^ T cells, ultimately resulting in T cell suppression after mid-gestation during pregnancy (52) (**Figure 2A**). The study offers evidence that B cells specific to a model trophoblast antigen are significantly suppressed via CD22–LYN inhibitory signaling (52). A complete understanding of the role of B cells as antigen-presenting cells during pregnancy is still elusive. Much of the current insights into this conundrum come from T-cell research in mouse models. Additional research in this area, including the effects of microchimerism on B cell function, is required to fully elucidate the complex interplay between maternal B cells and fetal antigens during pregnancy.

### 4.2 Antibody production

B cells serve a dual role within the immune system during pregnancy (**Figure 2A**); they produce protective antibodies contributing to a stable immune microenvironment at the maternal-fetal interface but may also generate autoantibodies that could disrupt pregnancy (**Figure 2**). The protective antibodies, also known as asymmetric antibodies (AAb), cannot form antigen-antibody complexes due to a structural irregularity involving an oligosaccharide residue (53, 54). These antibodies cannot trigger immune responses, potentially offering protection to the paternal antigens. Low maternal serum AAb levels in the first trimester are associated with spontaneous abortion later in pregnancy (55). The production of AAb can be influenced by IL-6, and progesterone-induced blocking factor (PIBF) (56–58). Estrogen and progesterone regulate the synthesis of PIBF and IL-6, suggesting that these steroids also play crucial roles in AAb production (59, 60).

Over the past several decades, the relationship between autoantibodies and pregnancy has been a subject of ongoing debate. Studies have shown that a significant number of non-organ-specific autoantibodies are linked to adverse pregnancy outcomes, such as RPL, intrauterine growth restriction, PE, and PTB. However, besides antiphospholipid (aPL) antibodies and anti-thyroid antibodies, many findings remain inconsistent. In patients with antiphospholipid syndrome (APS), an autoimmune condition characterized by the production of aPL, there is an increase in peripheral CD27^−^IgM^+^ naïve B cells, transitional B cells, CD19^+^CD5^+^ B cells, and plasmablasts, which are potential sources of autoreactive antibodies (61). Additionally, a decrease in IL-10-producing Bregs has been observed in the peripheral blood of patients with primary antiphospholipid syndrome (PAPS) and SLE with antiphospholipid syndrome (SLE/APS) (61). These B cell dysregulations may contribute to the pathogenesis of APS by disrupting the regulatory mechanisms that typically suppress the autoreactive B cell responses (61, 62). While most studies have focused on peripheral blood, the B cells in reproductive tissue during pregnancy have seldom been explored.

### 4.3 B cell secretion

At the maternal-fetal interface, B cells play a role in immune regulation through the secretion of cytokines. Key cytokines such as IL-10, IL-35, PIBF1, and transforming growth factor beta (TGF-β) are crucial in modulating immune responses and ensuring a successful pregnancy (**Figure 2A**).

IL-10 has anti-inflammatory properties. The IL-10-producing B cells can suppress Th1 and Th17 differentiation and prevent autoimmune disease development or alleviate established disease (63). These B cells can also inhibit T cells’ ability to generate tumor necrosis factor alpha (TNF-α) and IFN-γ. Additionally, the IL-10-producing B cells have longer contact times with the CD4^+^CD25^−^ T cells compared to IL-10-negative B cells, promoting the differentiation of these T cells into regulatory T cells (Tregs) (64).

B cells that produce and respond to IL-35 have been found in the uterine draining lymph nodes and spleens of pregnant mice, as well as in the peripheral blood of pregnant women. The function of these B cells is similar to that of the IL-10-producing B cells and contributes to maintaining immune tolerance during gestation (65–67). Serum IL-35 levels increase during a healthy pregnancy but decrease in cases of recurrent spontaneous abortion (68). One potential mechanism by which IL-35 operates during pregnancy is by regulating T-cell responses. B cell-produced IL-35 assists in controlling T cell-mediated autoimmunity by restricting the activation and proliferation of pathogenic T cells. In contrast, the absence of IL-35 enhances T cells responses against infections (69).

Besides IL-10 and IL-35, B cells can also secrete PIBF1 to help prevent pre-term labor in the later stages of pregnancy, an effect that is amplified by the therapeutic administration of interleukin 33 (IL-33) (39). PIBF1 protects normal pregnancy by reducing inflammation through the inhibition of pro-inflammatory cytokines and neutrophil activation, and by modulating immune responses, including the suppression of NK cell activity and support of B cell function (39). Furthermore, TGF-β produced by B cells plays a crucial role in fostering the development of Tregs. Specifically, TGF-β can cause dendritic cells to adopt a tolerogenic phenotype, supporting the expansion and maintenance of Tregs and contributing to an immune-tolerant environment (70). TGF-β is also part of a broader set of mechanisms through which B cells exercise regulatory functions, such as producing other cytokines like IL-10 and IL-35 (71).

### 4.4 Functional roles of B cell subsets

At the maternal-fetal interface, a unique immunological environment, various B cell subsets play vital roles in maintaining tolerance and immune protection. These subsets include B1 cells, and B2 cells (which encompass Bregs, transitional B cells, naïve B cells, memory B cells, MZ B cells, and plasma cells) (**Figure 1B**). Each subset exhibits distinct functions and mechanisms, contributing to the delicate balance between immune tolerance and protection in pregnancy (41, 46, 72–81). It is crucial to recognize that significant differences exist between B cells found in peripheral blood and those at the maternal-fetal interface. Due to the challenges associated with obtaining human tissue, most research focuses on peripheral blood B cells. Circulating B cell numbers are lower during pregnancy compared to non-pregnant women, possibly because they are concentrated at the maternal-fetal interface (36, 82).

#### 4.4.1 B1 cells

B1 cells are a subset of B lymphocytes crucial to humoral immune response. These cells predominantly reside in the peritoneal cavity of mice and peripheral blood in humans. Mouse B1 cells specifically express CD19, and B220, low levels of CD23 and IgD, and high levels of CD43 and IgM. Based on CD5 expression, the B1 cells can be categorized into the B1a (CD5^+^ cells) and B1b (CD5^-^ cells) subtypes (83). In humans, B1 cells are identified by the presence of CD20^+^CD27^+^CD43^+^CD70^-^ and may also express CD5^+^. There is ongoing debate regarding the identification and functional roles of human B1 cells compared to their mouse counterparts.

B1 cells contribute to the production of natural antibodies and autoantibodies in the serum, which are essential for regulating immune responses (84). Additionally, B1 cells participate in mucosal immunity by generating immunoglobulin A (IgA) plasma cells in the lamina propria, with CD5^-^ B1b cells potentially playing a crucial role in the mucosal IgA system (85). In older animals, B1 and B2 cells are implicated in B cell expansion and plasma cell accumulation. A homeostatic mechanism helps control the deregulated growth of B cells in aged mice. B1a cells, in particular, have been linked to dysregulated growth and tumor development. Studies suggest that peritoneal B1a cells consistently express the Stat3 oncogene, which may predispose them to oncogenesis due to their unique proliferation (86).

B1 cells have a complex role in pregnancy involving a delicate balance between their protective roles and potential for complications. During pregnancy, B1a B cells with high expressions of plasma cell alloantigen 1 (PC1) are present in the mouse peritoneal cavity. These cells generate regulatory substances such as IL-10, which contribute to immune tolerance and safeguard the fetus from maternal immune rejection. This process is achieved by influencing the balance between different T cell populations. Conversely, the transfer of B1a B cells with low PC1 levels has been shown to increase the likelihood of fetal rejection in pregnant mice (87). Damián Oscar Muzzio et al. demonstrated that B1a B cells, found in the mouse peritoneal cavity, have lower CD86 expression during normal pregnancy and suppress the differentiation of pro-inflammatory Th1 and Th17 cells. In contrast, during instances of pregnancy complications, these B1a B cells retain higher CD86 expression levels and promote Th1 and Th17 cell differentiation (74).

Disruptions in B1 cell function or numbers have been linked to pregnancy complications. B1 cells can also produce autoantibodies that target maternal or fetal tissue, causing PE or recurrent spontaneous abortion. These autoantibodies may disturb the critical immune balance needed for a healthy pregnancy (72). It is important to recognize that the precise mechanisms and effects of B1 cells on pregnancy are still under investigation.

#### 4.4.2 Regulatory B cells

In both mice and humans, the Bregs exhibit a variety of markers, including CD21^hi^CD23^+^IgM^hi^ (T2-MZP) B cells, TIM-1^+^ B cells, CD9^+^ B cells, CD5^+^ B1a cells, GIFT15 B cells, and CD44^hi^CD138^+^ plasmocytes (88). Human Bregs are present in peripheral blood, lymphoid tissues, and the uterus, whereas in mice, they reside in the spleen, lymph nodes, and bone marrow (79, 89).

The primary function of Bregs is to regulate immune responses. They produce anti-inflammatory cytokines, such as IL-10, which suppresses excessive immune reactions and maintains immune balance. This action is vital in preventing autoimmune diseases, where the immune system mistakenly targets the body’s cells, and in managing chronic inflammation (90). Furthermore, Bregs control inflammation during infections, ensuring an effective immune response without causing extensive damage to host tissues (91). They also influence other immune cells, including T cells, dendritic cells, and macrophages, through direct cell-to-cell interactions or cytokine secretion.

During pregnancy, Bregs in fetal-maternal interface play a vital role in promoting maternal-fetal tolerance. Along with their functions in the periphery, they also release immunosuppressive cytokines like IL-10, IL-35, and TGF-β1 to reduce inflammatory responses and create a tolerogenic environment (89, 92). They also contribute to tissue remodeling essential for placental development (93). Moreover, Bregs in the uterus or circulation can interact with immune cells, such as T cells, to encourage the development of Tregs and suppress the functions of effector T cells, preventing pregnancy complications (79). Bregs promote the expansion and function of Tregs primarily through the secretion of IL-10 and TGF-β, which create an immunosuppressive environment that enhances the proliferation and stability of Tregs. Additionally, Bregs interact directly with Tregs via cell surface molecules such as CD40 and CD86, further supporting their expansion and suppressive functions. Human peripheral Bregs suppress effector T cell functions through a combination of cytokine production, expression of inhibitory surface markers, induction of Tregs, modulation of antigen-presenting cells, production of proapoptotic molecules, and TLR interactions (94, 95).

#### 4.4.3 Transitional B cells

Transitional B cells in mice and humans are characterized by the expression of specific surface markers CD24 and CD38 (96). They are located in peripheral lymphoid organs, such as the spleen and lymph nodes, where B cells undergo maturation. As the transitional B cells exit the bone marrow, they are tested for autoreactivity (97). If they exhibit strong binding to self-antigens, they are typically eliminated or in specificity through receptor editing. Conversely, if transitional B cells recognize foreign antigens with adequate affinity, they are allowed to continue maturing. This mechanism ensures an effective immune response against pathogens while protecting the body’s tissues (98). As a result, the transitional B cells help establish central tolerance by eliminating self-reactive B cells and preventing autoimmunity (99). Moreover, the transitional B cells suppress autoreactive CD4^+^ T cell proliferation, inhibit pro-inflammatory T cell differentiation, promote the conversion of effector T cells into Tregs, and suppress CD8^+^ T cell responses (99).

The role of transitional B cells in pregnancy is yet to be completely understood. As pregnancy progresses, the number of decidual transitional B cells increases, suggesting a protective role in fetal tolerance and immune regulation (78). In contrast, the percentage of transitional B cells in peripheral blood reduces during pregnancy (100). Furthermore, human circulating transitional B cells in the third trimester of pregnancy are capable of producing anti-inflammatory cytokines, such as IL-10, which are crucial for maintaining pregnancy (82).

#### 4.4.4 Naïve B cells

In mice, naive B cells are mainly identified by their surface expression of IgM and IgD. They are primarily located in the bone marrow and secondary lymphoid organs, including the spleen and lymph nodes. Human naive B cells, on the other hand, are marked by IgD and CD27 and are produced in bone marrow and subsequently migrate to the peripheral blood and secondary lymphoid organs (96).

Naive B cells initiate the primary immune response and act as the first line of defense for the adaptive immune system upon an initial pathogen encounter (101). Each naive B cell possesses a distinct BCR, enabling it to recognize a broad range of pathogens. Some naive B cells exhibit SHM to enhance their BCRs’ affinity for antigens. When an antigen is encountered, naive B cells activate and differentiate into either plasma cells, which produce antigen-specific antibodies, or memory B cells for long-term immunity (102). Although naive B cells initially produce IgM antibodies, they may transition to other antibody classes, such as immunoglobulin G (IgG), IgA, or immunoglobulin E (IgE), through CSR (103).

During pregnancy, naive B cells can be localized in the placental intervillous blood, a region within the placenta containing maternal blood. The interaction between CCL20, which is produced by trophoblasts and decidual stromal cells, and CCR6, which is present on mature naive B cells, enables the movement of these B cells toward the intervillous blood areas within the placenta (46). These mature naive B cells serve as guardians against pathogens, protecting both the mother and fetus from infections by producing natural antibodies (IgM) in a T-cell independent manner (46). These natural antibodies, which are generated without previous exposure to antigens, can identify and attach themselves to PAMPs and modified self-antigens. This ability enables them to provide an immediate defense against infections, promote the removal of dying cells, manage inflammation, and exhibit anti-cancer properties (104).

#### 4.4.5 Memory B cells

Memory B cells are situated in various locations, including the spleen, lymph nodes, bone marrow, and peripheral blood of both mice and humans. Their primary role is to remember past infections, enabling the body to launch a faster and more effective immune response when encountering the same pathogen again. After an initial infection or vaccination, the memory B cells can remain in the body for years or even decades. If the same germ invades again, these cells can rapidly transform into plasma cells, producing high-affinity antibodies to neutralize the germ. Thus, memory B cells are vital in immunological memory, offering long-term protection and enabling secondary immune responses (101, 105). In humans, CD27 is a surface marker commonly used for the memory B cells. However, not all memory B cells express CD27 (96, 106). The discovery of other markers, such as PD-L2, CD80, and CD73, has unveiled considerable phenotypic diversity of memory B cells, leading to the identification of at least five unique subsets of these cells (107). Interestingly, Marilen Benner et al. discovered that a subset of memory B cells increases in the decidua during pregnancy (41).

During pregnancy, the primary function memory B cells is to assist the mother’s immune system in tolerating the fetus by avoiding the initiation of a strong immune response against fetal antigens. In addition, human circulating memory B cells are prepared to combat infections that may endanger the mother or fetus (108). B cells can identify paternal antigens in the fetus and placenta that are foreign to the mother’s immune system, such as specific Human Leukocyte Antigen (HLA) molecules. The memory B cells generated during a woman’s first pregnancy can influence her immune response in later pregnancies. If these cells encounter the same paternal antigens in future pregnancies, they can promote faster immune recognition and tolerance. It is noteworthy that uterine-resident B cells may not be involved in the production of alloantibodies. Instead, B cells located outside the uterus predominantly differentiate into plasma cells to produce antibodies (109, 110). Acquiring an in-depth understanding of memory B cells’ functions and regulation during pregnancy is essential for addressing complications like spontaneous abortion, PE, and other immune-related pregnancy issues. This knowledge also bears significance for organ transplantation, as the memory B cells specific to HLA antigens can affect transplant acceptance and rejection.

#### 4.4.6 Marginal zone B cells

MZ B cells form a unique subset of B cells located primarily in the spleen’s marginal zone. The strategic position allows them to play a vital role in immune responses, particularly in rapidly responding to blood-borne pathogens. MZ B cells can initiate an immune response more quickly than other B cell subsets due to their direct antigen recognition capability, bypassing the need for antigen processing and presentation by other cells. Once an antigen is captured, MZ B cells initiate an immune response by transforming into plasma cells that generate antibodies. They can also present antigens to T cells, triggering a customized adaptive immune response. Additionally, MZ B cells help maintain immune memory, as they can swiftly respond to previously encountered antigens for a potent secondary immune response.

Human and mouse MZ B cells exhibit a distinct set of surface markers. In mice, MZ B cells are marked by a high expression of CD21/CD35 and typically low or negative for CD23, differentiating them from the FO B cells, which exhibit high CD23 expression. Compared to the FO B cells, MZ B cells express higher levels of CD21, CD1d, CD38, CD9, and CD25. Human MZ B cells are generally CD27^+^, a marker shared with memory B cells. Similar to mice, human MZ B cells express high levels of IgM and CD21, and low levels of IgD. Additionally, CD1c is present in human MZ B cells (96, 111).

During pregnancy, the immune system of mice is suppressed to promote fetal tolerance by inhibition of differentiation of T cell-dependent FO B cells, which generate highly specific IgG antibodies. To counterbalance this suppression, the T cell-independent MZ B cells from mouse spleen or lymph nodes form short-lived plasma cells producing non-specific antibodies (IgM/IgA) that protect the mother from infections during pregnancy and minimize the likelihood of producing autoreactive B cells that target fetal tissues (112, 113).

#### 4.4.7 Plasma cells

In both mice and humans, plasma cells are generally recognized by CD138, CD27, and CD38 (114). They are predominantly located in the bone marrow, where they generate and release antibodies. During an immune response, plasma cells may be found in secondary lymphoid organs, including the spleen and lymph nodes (115). These cells are the main producers of antibodies that defend against pathogens by attaching to them, obstructing their entrance into host cells, and triggering the complement cascade. Moreover, the antibodies can coat pathogens, improving their recognition by other immune cells, such as macrophages and neutrophils, which then engulf and eliminate the pathogens (114). Certain plasma cells also secrete cytokines like IL-35 and interleukin 17 (IL-17), which modulate the activity of other immune cells (116).

Plasma cells in the placenta are vital in maintaining immune tolerance during pregnancy by producing specific antibodies that interact with the Fc receptors on various immune cells. This interaction results in suppression of inflammatory responses and promotion of tolerance (117). IgG antibodies can also cross the placenta, providing the fetus with passive immunity against infections and supporting healthy fetal development (118, 119). Additionally, these antibodies can be found in breast milk, offering passive immunity to the infant after birth (120).

For pregnant women with SLE, modulation of circulating plasma cell function can lead to reduced disease severity due to shifting of the immune system towards a more regulatory and less inflammatory state. However, in SLE patients with pregnancy complications, certain plasma cell-related transcriptomic modules may not be downregulated to the same degree as in healthy or uncomplicated SLE pregnancies. This inadequate downregulation may contribute to the persistence or worsening of autoimmune activity (36, 121).

## 5 Hormonal regulation of B cell development and functions

Hormonal regulation of B cell development and function is a complex process that involves a multitude of hormones and signaling pathways. A notable difference is observed between the genders, with females typically exhibiting higher levels of immunoglobulins IgM and IgG than males, which is thought to be due to the influence of sex hormones (122). Hormonal regulation not only maintains standard immune responses but also enables the immune system to adapt to the evolving physiological conditions throughout pregnancy. Pregnancy-related hormones, such as human chorionic gonadotropin (hCG), progesterone, and estrogen, are essential for a successful pregnancy (**Table 1**) (123, 124). Each hormone has a specific role: hCG supports the corpus luteum in the ovary and prompts it to produce progesterone, which prepares the uterus for pregnancy and suppresses the immune response to avoid embryo rejection. Concurrently, estrogen assists in modulating the mother’s immune system and promotes fetal growth (125).

**Table 1:** The roles of key hormones in B cell development and function during pregnancy.

Hormones	Secreted organ	Process	Effects	References
**Estrogens**	Ovaries, placenta	Development	Pro-B, Pre-B, immature B ↓ mature B cells ↑ memory B cells ↑ Plasma B cells ↑ Plasmablasts ↑ Bregs ↑	(142–145)
		Activation	activation-induced deaminase (AID) ↑ CD80 ↓	(146, 147)
		Survival	Apoptosis ↓	(146)
		Antibodies	IgA, IgG1,IgG2 IgG3 and IgE, anti-DNA antibodies↑	(144, 148)
**Progesterone**	Ovaries, placenta	Development	Pro-B, Pre-B, immature B ↓ Plasma B cells ↓/↑ Plasmablasts ↓	(135, 145, 149)
		Activation	BAFF ↓ CD80 and CD86 ↓ activation-induced deaminase (AID) ↓	(135, 147, 150)
		Survival	Apoptosis ↑	(134)
		Antibodies	IgA, IgG ↑	(135)
**Human Chorionic Gonadotropin (hCG)**	Placenta	Development	B1 cells ↑ Bregs ↑ plasma cells ↓	(73, 76, 137, 141)
		Activation	/	
		Survival	/	
		Antibodies	IgG ↑	(137)

symbols ↓: Decrease in expression. symbols ↑: Increase in expression.

In humans, progesterone levels increase following ovulation and stay elevated throughout pregnancy. By interacting with specific receptors, progesterone efficiently suppresses inflammatory immune responses and initiates tolerance pathways. Simultaneously, estrogen concentrations consistently rise until term, modulating immune cell populations and functions to support fetal tolerance through estrogen receptor alpha (ERα) and estrogen receptor beta (ERβ). During early pregnancy, hCG levels rapidly escalate, reaching their peak between 9-12 weeks of gestation (125, 125). This hormone serves a vital function in stimulating progesterone production, which in turn assists in maintaining the pregnancy. In combination, these hormones work together to regulate the immune system during pregnancy, fostering a favorable environment for fetal growth.

Estrogen has been demonstrated to have suppressive effects on B cell lymphopoiesis in bone marrow, specifically hindering the differentiation from pro-B cells to pre-B cells and consequently leading to a reduction in B cell precursors (126). This suppressive action is mediated through both direct effects on B cells and indirect effects on stromal cells (127). It is partially attributed to decreased production of the homeostatic cytokine interleukin-7 (IL-7) and increased expression of soluble frizzled-related protein 1 (sFRP1) by bone-lining stromal cells (128, 129). The observed decline in B cell production during pregnancy is suggested to serve as a protective mechanism against the development of autoimmune diseases.

On the other hand, estrogen enhances B cell survival by elevating the expression of anti-apoptotic proteins, such as bcl-2, and fostering the differentiation of B cells into antibody-generating plasma cells. This process leads to an increase in antibody production, particularly IgG and IgA. Estrogen also enhances CSR and SHM in mature B cells, mechanisms that expand antibody diversity and affinity. This is achieved by directly stimulating the transcription and function of activation-induced deaminase (AID), a vital enzyme involved in these processes that are crucial for adaptive immunity (130). Moreover, estrogen affects the formation of Bregs, contributing to the establishment of an immunosuppressive environment necessary for a successful pregnancy (131).

Progesterone affects B cells through both genomic and non-genomic mechanisms by interacting with progesterone receptors (PR-A and PR-B) present on B cells. It modulates B cell development in the bone marrow by decreasing the amount of BAFF, a key cytokine for B cell maturation and survival. Progesterone can also hinder CSR and SHM of B cells by reducing AID mRNA levels. This is accomplished through binding to the promoter region of the AID gene, which leads to the assembly of an inhibitory transcription complex (132). This inhibition changes the Ig glycosylation patterns and affects the immune response, contributing to the alleviation of autoimmune diseases during pregnancy. The modified glycosylation patterns can reduce the inflammatory potential of antibodies, leading to a more regulated immune response (132, 133). Progesterone also promotes the production of Bregs that generate immunosuppressive cytokines like IL-10 and TGF-β. Moreover, progesterone has been observed to increase the apoptosis of certain B cell lines and suppress the activation of B cells by reducing the expression of co-stimulatory molecules such as CD80 and CD86 on B cells, as seen in an *in vitro* study (134, 135).

The combined effect of progesterone and estrogen enhances humoral immunity during pregnancy by controlling the differentiation and growth of specific B cell subsets, such as memory B cells and plasma cells, as well as modulating Ig production, particularly IgG, through influencing Tfh cells and B cell interactions (136). The interplay between Tfh cells and B cells is vital for generating high-affinity antibodies and establishing immunological memory (26). These hormones boost the activity and functionality of Tfh cells, which in turn offer essential support to B cells, such as providing signals for B cell activation, survival, and differentiation into antibody-producing plasma cells or memory B cells (124).

HCG can bind to the luteinizing hormone/choriogonadotropin receptor (LHCGR) found on B cells, subsequently initiating signaling pathways that influence B cell proliferation. Higher hCG levels have been detected in the serum of PE patients compared to normal pregnancies. The persistently elevated hCG levels appear to stimulate the expansion of CD19^+^ CD5^+^ cells in peripheral blood through its receptor, which is highly expressed in these cells. *In vitro* exposure to hCG leads to significant proliferation of CD19^+^ CD5^+^ cells. The increased presence of these B cells, capable of producing autoantibodies such as angiotensin II type 1 receptor autoantibodies (AT1-AA), may contribute to the autoimmune-like symptoms observed in PE (73). In contrast, hCG can raise the levels of asymmetrically glycosylated IgG antibodies, an unusual structure of IgG molecules that protect pregnancy by reducing alloreactive immune responses (137–139). In addition, hCG triggers the conversion of CD19^+^ cells into Bregs in peripheral blood, similar to the effect of estrogen, contributing to pregnancy tolerance (76). It can also promote pregnancy success by enhancing the function of human Bregs for fetal survival (76). Interestingly, hCG has been found to inhibit the differentiation of murine splenic B cells into plasma cells, suggesting that hCG may regulate the function of different B cell subsets variably during pregnancy (140, 141).

In summary, hormonal regulation during pregnancy greatly affects B cell development and functionality. Key hormones such as estrogen, progesterone, and hCG modulate B cell proliferation, survival, antibody production, and the balance of various B cell subsets. This hormonal regulation ensures the immune system appropriately adapts to the distinct demands of pregnancy, maintaining a fine balance between immune tolerance and protection against infections.

## 6 B cells and pregnancy complications

B cells have been associated with the development of various pregnancy complications, such as PE, gestational diabetes mellitus (GDM), PTB, and RPL (**Figure 2B**).

### 6.1 Preeclampsia

PE is a common gestational disease affecting 2-5% of all pregnancies and is the leading cause of prenatal mortality and morbidity. PE is also associated with a high incidence of fetal growth restriction, and short- and long-term maternal and perinatal complications, making it a significant burden on the healthcare system. The etiology of PE is linked to defective trophoblast differentiation and functions, leading to abnormal placental development, high blood pressure, insufficient placental perfusion, and maternal-fetal exchange defects. Impaired fetus-maternal tolerance and altered immune homeostasis are widely believed to be significant factors in the development of PE (151). The systematic immune system is activated while the immunoregulatory system is suppressed in women with PE (152, 153). When activated by AT1-AAs in PE, the anti-angiotensin II type 1 (AT1) receptor, which is expressed by endothelial cells, vascular smooth muscle cells, cardiac myocytes, and renal cells, enhances immune responses by increasing the production of pro-inflammatory cytokines, promoting oxidative stress, and contributing to endothelial dysfunction, thereby disrupting immune homeostasis (151). B cells, with the help of T cells, are thought to produce antibodies that activate the immune system and target the AT1 receptor in women with PE (151). These antibodies can also form immune complexes (ICs) when they bind to target antigens, including those from fetal trophoblasts, which serve as a natural antigenic source during normal pregnancy. Although ICs are present in normal pregnancies, their elevated levels and altered composition in PE can trigger inflammatory responses through circulation or tissue deposition. This may contribute to the inflammatory response and endothelial dysfunction characteristic of the condition (154, 155).

Ai-Hua Liao et al. investigated the functional changes in human peripheral B cells in women with PE compared to healthy ones. The study found that women with PE had a higher percentage of memory B cells (CD27^+^CD38^-^) and plasma cell precursors (CD27^+^CD38^+^). Additionally, irrespective of stimulation, the mean percentages of generated plasma cells were significantly higher in the PE group. There were also more antibody-producing cells in women with PE following activation. These findings suggest that the functional changes in circulating B cells may contribute to the etiology of PE (156). Federico Jensen et al. found that the frequency of CD19^+^CD5^+^ B1a B cells was significantly higher in the peripheral blood of preeclamptic patients compared to normal pregnant women. These CD19^+^CD5^+^ B cells were identified as the source of AT1-AAs and found in the placenta of preeclamptic patients but are almost absent in normal pregnancies. Additionally, the elevated levels of hCG in the serum and placenta supernatant of these patients drive the increase in CD19^+^CD5^+^ B cells. Approximately 95% of these cells express the hCG receptor and expand upon hCG stimulation, which further promotes the production of pathogenic autoantibodies (73). In animal models of PE, depletion of maternal B cells with rituximab reduces blood pressure and increases fetal weight (157). Reducing the B cell numbers decreases the AT1-AA levels and alleviates PE symptoms (158). Kristin Malinowsky demonstrated that the *in vivo* transfer of B1a B cells, which were strongly activated by progesterone, led to the accumulation of deposits in the mothers’ kidneys, resulting in kidney damage (159). This finding highlights the significance of the B cells in PE pathogenesis. Since PE is a complex condition with poorly understood mechanisms, further research is crucial to fully comprehend B cells’ contribution to PE.

### 6.2 Gestational diabetes mellitus

GDM is a condition in which women with no prior history of diabetes exhibit high blood glucose levels during pregnancy, which usually resolve after delivery (160). It is the most prevalent medical complication in pregnancy. The risk factors of GDM include maternal obesity, advanced maternal age, a history of GDM, a family history of type 2 diabetes, and certain ethnic backgrounds. GDM can lead to complications such as hypertensive disorders in the mother, excessive fetal growth and adiposity, and increased long-term risks of diabetes, obesity, and cardiovascular disease for both mother and child (160). GDM is associated with a relative insufficiency in insulin response or β-cell defects that cannot compensate for the increased insulin resistance during pregnancy (161). The significance of B cells in GDM is shown by a positive correlation between the percentage of B cells in peripheral blood and insulin resistance (162), and an increased proportion of adipose tissue B cells in pregnant women with GDM, particularly in those who produce pancreatic autoantibodies (163, 164). These finding suggests that the B cells may serve as a predictor for insulin resistance in women with GDM. Despite these observations, the exact role of B cells in GDM remains uncertain. Further research is needed to clarify the contribution of B cells to the pathophysiology and development of GDM.

### 6.3 Preterm birth

PTB refers to the delivery of a baby before 37 weeks of gestation. PTB affects approximately 11% of births worldwide, with significant variations due to differences in gestational age measurement and definitions (165–167). Major risk factors for PTB include socio-demographic, nutritional, medical, obstetric, and environmental factors, although most PTB cases occur without clear risk factors. Complications of PTB often involve increased risks of neurodevelopmental impairments, respiratory issues, and gastrointestinal complications, contributing significantly to perinatal morbidity and mortality (168). There is a significant increase in B cells, particularly the B1 cells, in the decidua of women experiencing preterm labor compared to term labor (78). In a mouse model of LPS-induced PTB, significant increases in IL-33 receptor-expressing B1cells in the spleen and decidual B cells are observed during the acute phase, indicating their critical role in the immune mechanisms underlying PTB (169). There is also increased PR-A expression in B cells among women with PTB, along with elevated levels of pro-inflammatory cytokines such as IL-6, IL-21, and TNF-α, particularly in cases of spontaneous PTB and PE/HELLP syndrome. This correlation suggests a pronounced inflammatory response preceding PTB, emphasizing the potential of PR-A^+^ B cells as biomarkers for predicting PTB risk (170).

### 6.4 Recurrent pregnancy loss

RPL is characterized by the failure of two or more clinically recognized pregnancies before reaching 20-24 weeks of gestation, encompassing both embryonic and fetal losses (171). RPL affects approximately 2-5% of couples attempting to conceive (172). RPL can have significant psychological impacts on the affected couples, including grief, anxiety, and depression, and may increase risks of future pregnancy complications (171). RPL has been associated with parental chromosomal abnormalities, maternal thrombotic diseases, endocrine disorders, and immune dysfunction (171, 172). Autoantibodies produced by B cells are associated with RPL (173). The absolute count of non-switching memory B cells from peripheral blood is significantly elevated in women with RPL (14). Moreover, the endometrial B cell levels are higher in women with RPL compared to their healthy counterparts (174). The production of IL-10 by B cells from human peripheral blood, as well as overall IL-10 levels, are lower in women with RPL. And a negative correlation exists between the proportion of these IL-10-producing B cells and serum autoantibody levels in the RPL patients (175). Although there is no relevant human data, the abortion rate in mouse models with a tendency for miscarriage (CBA/J×DBA/2J) decreased after exogenous administration of regulatory B10 cells (138). These findings suggest that RPL may be linked to abnormal maturation and activation of B lymphocytes.

## 7 B cells and autoimmune: implications for pregnancy and target therapies

The function of B-cells during pregnancy is complex and multifaceted. The development of B cell-targeting therapies offers a promising opportunity for preventing and treating pregnancy complications. Nonetheless, this approach necessitates a more profound comprehension of the delicate equilibrium between immune tolerance and response throughout pregnancy.

Autoimmune diseases are characterized by dysregulated immune tolerance, which is similar to pregnancy complications. It is worth investigating whether drugs approved for autoimmune diseases could be repurposed for treating pregnancy complications. B-cell dysregulation has been identified as a critical factor in pregnancy-related autoimmune diseases, particularly in conditions such as APS, SLE, and autoimmune thyroid disease (AITD) (176–180). In these disorders, autoreactive B cells produce pathogenic autoantibodies that directly affect pregnancy outcomes. For instance, in APS, B cells generate aPL antibodies that target phospholipid-binding proteins on placental tissues, potentially leading to thrombosis, placental insufficiency, and fetal loss (170). In SLE, autoantibodies such as anti-Ro and anti-La can cross the placenta, possibly causing neonatal lupus and congenital heart block (181). In AITD, thyroid peroxidase (TPO) and thyroglobulin (Tg) antibodies may also cross the placenta, potentially interfering with maternal and fetal thyroid function (182). The discovery of TPO expression in the endometrium and placenta suggests that these antibodies may directly impact reproductive tissues. These antibodies have the potential to cause local damage, activate inflammatory responses, and disrupt local thyroid hormone production, thereby creating an unfavorable environment for embryo implantation and fetal development (177). Furthermore, Lee et al. showed that TPO antibodies might bind directly to embryos (183). B cells further contribute to pregnancy complications through cytokine production, with increased levels of pro-inflammatory cytokines, such as IFN-γ, TNF-α, and IL-6, which promote placental inflammation and dysfunction (179, 184). B cells can also present autoantigens to T cells, activating them and further intensifying the autoimmune response at the maternal-fetal interface (185). The formation of immune complexes by autoantibodies may trigger complement activation, leading to placental damage and hindered fetal development. Various B cell subsets, including naïve, memory, plasma, and MZ B cells, participate in these processes, while Bregs function to suppress inflammation through IL-10 and TGF-β production (89, 93). This intricate interplay of B cell-mediated mechanisms may result in various pregnancy complications, such as RPL, PE, intrauterine growth restriction, and PTB in women with autoimmune diseases (180).

B cell-targeted therapies have become a significant approach in treating various autoimmune diseases. These encompass several strategies such as: 1) direct depletion using monoclonal antibodies like rituximab, ocrelizumab, and obinutuzumab; 2) indirect depletion by blocking cytokines essential for B cell survival, such as belimumab targeting BAFF; 3) blockade of the co-stimulatory signal with CD40/CD40L antibody; 4) B cell modulation using agents like Bruton Tyrosine Kinase (BTK) inhibitors, Ibrutinib, and acalabrutinib; 5) targeting plasma cells with anti-CD38 antibody daratumumab (179, 186, 187). While first-generation therapies like rituximab have been widely used, second-generation agents and newer approaches, including targeting CD19, proteasome inhibition, and chimeric antigen receptor (CAR) T-cell therapy, have shown improved efficacy in certain conditions (186, 187). These treatments have had varied success across diseases like rheumatoid arthritis, SLE, multiple sclerosis, and ANCA-associated vasculitis, with their effectiveness often depending on the specific disease mechanism and the balance between pathogenic and protective B cell functions (186). In pregnancy, current therapies for B cell-mediated autoimmune disorders focus on managing symptoms and reducing risks, including the cautious use of B cell depletion therapy, intravenous immunoglobulin, hydroxychloroquine, and anticoagulants for conditions like APS (180, 188–191). Future research efforts to develop more targeted and safer treatments, including new B cell-specific therapies, enhancement of regulatory B cell function, development of antigen-specific immunotherapies, and investigation of cytokine-targeted approaches, are needed. There’s also growing interest in microbiome modulation, nanoparticle-based drug delivery systems, and gene editing technologies (192–194). Researchers are working to identify biomarkers for personalized treatment and conduct long-term safety studies, with the ultimate goal of developing more effective, tailored treatments that can modulate the immune system with minimal side effects, thus maintaining maternal health while ensuring optimal fetal development.

## 8 Conclusions and perspectives

In conclusion, the current lack of research on the role B cells play during pregnancy indicates a valuable area for future investigation. Future studies should concentrate on the roles of different B cell subsets, their regulation by hormones and cytokines, and potential therapeutic targets for pregnancy-related disorders. This includes understanding the dynamics of B cells at the maternal-fetal interface and their interactions with other immune cells, such as T cells, NK cells, and DCs. Moreover, the impact of B cell-derived factors, including cytokines, chemokines, and antibodies, on pregnancy outcomes should be explored. Enhancing our understanding of B cells in pregnancy could lead to better strategies for improving maternal and neonatal health.

The role of B cells in pregnancy is critical but underexplored. These cells balance immune tolerance for successful gestation and defense against pathogens. Despite their significance, the specific contributions of B cells to pregnancy outcomes, including complications like PTB and PE, remain unclear. This article overviews the current understanding of B cells’ multifaceted functions at the maternal-fetal interface, emphasizing their dual role in creating a favorable environment for fetal development while also being implicated in various pregnancy-related disorders. Bregs and their secretion of IL-10 are crucial in maintaining tolerance towards the fetus, showcasing the body’s sophisticated mechanism to prevent immune responses against the semi-allogeneic fetus. In contrast, the production of autoantibodies by certain B cell subsets highlights the complexity of immune regulation, where abnormalities can result in adverse pregnancy outcomes. The paper also emphasizes the significant hormonal regulation of B cell function during pregnancy. These findings suggest that the immune system’s adaptability during pregnancy is not only a response to the fetus’s presence but also to the hormonal environment, which modulates B cell activity and consequently influences pregnancy outcomes.

This review paper also explores the specific types of B cells at the maternal-fetal interface and clarifies their unique roles. The presence of naive B cells, memory B cells, and plasma cells emphasizes the immune system’s readiness to respond to pathogens, ensuring the fetus’s protection while maintaining tolerance. Although significant progress has been made in understanding B cell subsets and their activities during pregnancy, many questions still remain. It is important to recognize that identifying B cell subpopulations and their characteristics and roles during pregnancy, particularly within the maternal-fetal interface, is an area of interest because the current identification of these subpopulations is primarily based on studies of human peripheral blood and mouse models. Filling this knowledge gap could have substantial implications for both maternal and fetal health.

A new subset of B lymphocytes, termed age-associated B cells (ABCs), characterized by the expression of CD11c^+^CD19^+^B220^+^, has been identified and is associated with infection, aging, and autoimmune disease. Additionally, the long-term effects of B cell dysregulation during pregnancy on both maternal and offspring health should be investigated. This research will help determine if abnormal B cell function during pregnancy has lasting consequences for the health of both the mother and her child and may identify potential targets for interventions to improve long-term health outcomes. By addressing these gaps in knowledge, future research can offer valuable insights into the complex immune regulation that occurs during pregnancy and may lead to the development of novel therapeutic approaches to enhance maternal and fetal health.

The discussion on pregnancy complications and the involvement of B cells provides a new perspective on the pathogenesis of these conditions. The relationship between increased B cell activity, especially in the context of autoantibody production, and the development of conditions such as PE and RPL, highlights the need for further research. Unraveling the molecular mechanisms by which B cells contribute to these complications could lead to the development of innovative therapeutic interventions aimed at modulating B cell activity to prevent or alleviate these conditions. Consequently, potential therapeutic strategies might focus on B cells, including B cell depletion, modulation of B cell function (inhibiting or activating BCR signaling, targeting the co-stimulatory signal required for B cell activation), targeting specific antibodies, and enhancing the function of Bregs. However, due to the unique nature of pregnancy, the safety, timing, and dosage of intervention require careful and comprehensive evaluation.
